# Mitochondrial DNA: Inherent Complexities Relevant to Genetic Analyses

**DOI:** 10.3390/genes15050617

**Published:** 2024-05-12

**Authors:** Tomas Ferreira, Santiago Rodriguez

**Affiliations:** 1 Bristol Medical School, University of Bristol, Bristol BS8 1UD, UK; 2 Department of Clinical Neurosciences, School of Clinical Medicine, University of Cambridge, Cambridge CB2 0SL, UK; 3 MRC Integrative Epidemiology Unit, Population Health Sciences, Bristol Medical School, University of Bristol, Bristol BS8 1QU, UK

**Keywords:** mitochondrial DNA, genetic analyses, heteroplasmy, copy number variations, epigenetic modifications, genome-wide association studies, phenome-wide association studies, population genetics

## Abstract

Mitochondrial DNA (mtDNA) exhibits distinct characteristics distinguishing it from the nuclear genome, necessitating specific analytical methods in genetic studies. This comprehensive review explores the complex role of mtDNA in a variety of genetic studies, including genome-wide, epigenome-wide, and phenome-wide association studies, with a focus on its implications for human traits and diseases. Here, we discuss the structure and gene-encoding properties of mtDNA, along with the influence of environmental factors and epigenetic modifications on its function and variability. Particularly significant are the challenges posed by mtDNA’s high mutation rate, heteroplasmy, and copy number variations, and their impact on disease susceptibility and population genetic analyses. The review also highlights recent advances in methodological approaches that enhance our understanding of mtDNA associations, advocating for refined genetic research techniques that accommodate its complexities. By providing a comprehensive overview of the intricacies of mtDNA, this paper underscores the need for an integrated approach to genetic studies that considers the unique properties of mitochondrial genetics. Our findings aim to inform future research and encourage the development of innovative methodologies to better interpret the broad implications of mtDNA in human health and disease.

## 1. Introduction

Mitochondria and mtDNA display many particularities that distinguish them from the nuclear genome and are therefore relevant for methodological analyses of complex traits including structure, function, and human population history. Further, there are select aspects pertinent to mitochondria that warrant discussion, including inheritance, hypermutability, copy number, heteroplasmy, haplogroups, lack of introns, sensitivity to environmental factors, and epigenetics. Mitochondrial DNA has been thoroughly investigated since its discovery in 1963 and many aspects relevant to its inner workings have been elucidated [1]. Mostly, these have been investigated individually and lack a holistic approach.

Genetic studies, including genome-wide association studies (GWAS), epigenome-wide association studies (EWAS), phenome-wide association studies (PheWAS), pharmacogenomics, phylogenetic studies, and evolutionary studies, have become increasingly used for identifying associations between common DNA sequence variations and human traits. These studies are designed to identify associations between genotypes and phenotypes by examining differences in allele frequencies of genetic variants among individuals of similar ancestry but exhibiting distinct phenotypic characteristics [2].

Genetic studies have yielded significant advancements in our comprehension of the genetic factors underlying complex human traits. For instance, insights into conditions such as obesity, autoimmune diseases, schizophrenia, and Crohn’s disease have emerged from these studies [3,4,5,6,7]. Traditionally, GWAS have focused on identifying associations with single nucleotide polymorphisms (SNPs) on autosomal chromosomes. Recent advancements, however, are broadening the scope of data types used in these studies. This expansion includes the integration of sequencing-based GWAS methodologies, comprehensive gene-based analyses, and the inclusion of copy-number variation in the investigative repertoire [8]. However, the examination of genetic associations between the Y chromosome and mitochondrial DNA (mtDNA) has received comparatively less attention, with limited genetic studies conducted in these domains [9,10,11,12]. While GWAS, EWAS, and PheWAS play an increasingly important role in improving our understanding of mtDNA associations, they constitute only a portion of the extensive range of genetic studies this review aims to discuss.

We have recently reviewed the Y chromosome’s role in complex human traits [11]. Here, we aim to explore the evidence concerning mtDNA and its contribution to common traits. The investigation of mtDNA presents a complex and intricate locus for genetic analysis, marked by distinctive characteristics that require thorough examination. Through this review, we seek to explore the features of mtDNA that contribute to its complexity, discuss the achievements to date, and identify the challenges involved in conducting mtDNA genetic analyses within the context of common traits. 

## 2. Mitochondria and mtDNA

Mitochondria, from the Greek *mitos* and *khondros* (thread and grain-like), are double-membraned organelles found in all nucleated cells. They originate from a eukaryotic endosymbiont descended from α-proteobacterium [13]. Mitochondria contain their own genome (mtDNA).

The composition of mitochondria includes various subcompartments, each assuming an important role in cellular function. The outer mitochondrial membrane surrounds the organelle and regulates the passage of molecules in and out of the mitochondria. The inner mitochondrial membrane is highly folded into structures called cristae, which increase its surface area for ATP synthesis. The mitochondrial matrix, enclosed by the inner membrane, contains enzymes involved in the citric acid cycle and fatty acid metabolism [14].

The main role of mitochondria is related to energy metabolism, made possible by the OXPHOS complexes. In addition, mitochondria are essential in other cellular processes, including fatty acid oxidation, calcium homeostasis, ammonia and reactive oxygen species (ROS) detoxification, cholesterol synthesis, and apoptosis regulation [15,16,17]. It then follows that mitochondrial dysfunction could be associated with the variation of human complex traits. This has been reported in mitochondrial dysfunctions observed in conditions including Alzheimer’s disease, Amyotrophic Lateral Sclerosis, Huntington’s disease, and various types of cancer [18,19,20].

Table 1 shows the genes present within mitochondrial DNA. These encode tRNA molecules involved in translation, rRNA molecules responsible for peptide synthesis, and mitochondrial complexes. Variation in the genes encoding these complexes resulting in Mendelian traits has been thoroughly researched; in this review, we will focus on complex traits [21].

### 2.1. Structure of Human Mitochondrial DNA

**Figure 1:**
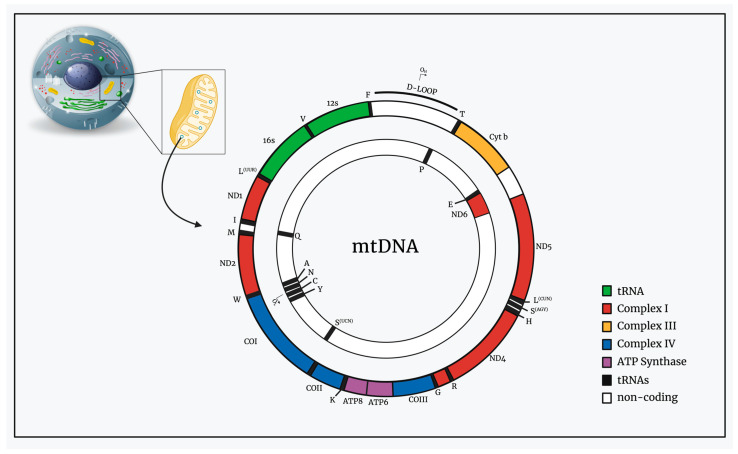
Schematic representation of mitochondria and the structure of mtDNA.

The mitochondrial genome is circular, double-stranded, and relatively small at 16 kb compared to the 3.3 billion bp nuclear genome [22]. The two strands can be distinguished by base composition: the imbalanced distribution of guanine results in the different buoyancies of each strand. This results in a heavy strand (h-strand) and a light strand (l-strand) (Figure 1) [23,24,25].

The heavy strand encodes the majority of the information, encoding 28 out of 37 genes. It comprises genes coding for two rRNAs (12S and 16S), 14 tRNAs, and 12 polypeptides. The l-strand codes for eight tRNAs and a single protein [26,27,28]. All of these 13 protein-encoding genes are essential constituents of the oxidative phosphorylation complexes (OXPHOS), responsible for energy production. Table 1 categorizes the seven subunits of complex I (*ND1*–*ND6*), the one subunit of complex III (Cyt b), the three subunits of complex IV *(COX I–COX III),* and the two subunits of complex V (*ATP6* and *ATP8*) [25].

This table categorizes the genes encoded on the mtDNA into those located on the heavy (H) strand and those on the light (L) strand. It includes the genes for ribosomal RNA (rRNA), transfer RNA (tRNA), and various polypeptides that are part of the respiratory chain complexes I, III, IV, and V. Each gene is identified by its mitochondrial nomenclature (e.g., MT-RNR1 for the 12s rRNA gene on the heavy strand).

#### 2.1.1. The Non-Coding Region and Displacement Loop

The mtDNA genome includes a 1.1 kb section known as the non-coding region (NCR). A significant portion of the NCR is a 655 bp long region known as the displacement loop (D-loop) [29]. One of the primary functions of the D-loop is serving as a promoter for both strands of mtDNA. Located strategically between the genes encoding tRNA^pro^ and tRNA^phe^, the D-loop initiates and regulates the transcription process, thereby playing a central role in the replication of mtDNA [29,30,31]. In addition to transcription regulation, it also plays an instrumental role in the production of core proteins for oxidative phosphorylation. Although mitochondrial replication occurs independently from nuclear DNA (nDNA) replication, it is worth noting that over 99% of the mitochondrial proteome is encoded in the nucleus, including all proteins required for mtDNA maintenance, replication, and transcription. There are 42 nuclear loci involved in the replication of mtDNA [32,33,34]. Moreover, the D-loop is also implicated in important roles in transcription and translation through specific mtDNA sequences [35,36,37].

Within the NCR, alongside the D-loop, are the most polymorphic regions of mtDNA, namely hypervariable segments I and II (HV1 at position 16,024–16,383 and HV2 at position 57–372). Genetic variants in these regions serve as mutational hotspots, and their role in the pathogenesis of complex human traits, including bladder, breast [38], lung [39], head and neck, colorectal, kidney, gastric, ovarian, and prostate tumors, has been identified (see Hertweck and Dasgupta, 2017 [40]). The inherent variability in these regions makes them particularly interesting for various applications, such as deriving haplogroups. Their analysis is commonly employed in forensic investigations for accurate identification purposes, and in population genetics studies to track evolutionary relationships and migration patterns. 

Due to its high mutation rate, the D-loop is a focal point in genetic studies, including GWAS. These mutations are important in deriving mitochondrial haplogroups, as discussed in Section 2.4.6, and are important for understanding hereditary patterns and the origins of certain diseases. Unlike nuclear DNA, the D-loop is unique to mtDNA, presenting particularities for the analysis of complex traits that are comparable only to the Y chromosome [11]. The high mutation rates of the D-loop also play an essential role in medical diagnoses, where they provide insights into various genetic diseases [41,42].

Another non-coding element within the mtDNA is the OriL, the origin of light strand replication. Situated away from the D-loop, OriL is responsible for the initiation of replication on the light strand of mtDNA [43]. Apart from this, mtDNA contains limited noncoding intergenic sequences [22]. In fact, the mitochondrial genome is compacted to such an extent that two coding sequences actually overlap (*MT-ATP6* and *MT-ATP8* as well as *ND4* and *ND4L*) [44].

#### 2.1.2. Reference Sequences

Since the first sequencing of the human mtDNA genome in 1981, the Cambridge Reference Sequence (CRS) has served as the standard for annotating mtDNA in fields such as molecular anthropology, forensic science, and medical genetics [22]. Recognizing the need for updates, the CRS was revised to the rCRS in 1999 [45]. While this reference sequence provides a convenient framework for cataloging mtDNA variations, it has been erroneously perceived as a ‘wild-type’ (WT) or consensus sequence by some medical geneticists. In a bid to address this misconception, Behar et al. in 2012 advocated for the adoption of the Reconstructed Sapiens Reference Sequence (RSRS) [46]. However, the RSRS, despite its accurate estimation, may not be a practical alternative to the rCRS as it blurs the distinction between ancestral lineage and the reference benchmark for human mtDNA [47]. The scientific community is at a juncture where it must critically evaluate and reach a consensus on reference sequence usage. Inconsistencies in reference sequence selection could hinder the comparability and generalisability of studies, as well as the validity of future analyses derived from the same datasets, thus potentially diminishing their impact, including GWAS based on mtDNA sequence data. Since the introduction of RSRS, there have been no significant updates in reference sequence development, signifying a need for continued discussion and potential revision in this area [48].

### 2.2. Function of Human Mitochondrial DNA

#### 2.2.1. The Functional Role of Human mtDNA

Mitochondrial complexes have been thoroughly characterized, both biochemically and genetically. The biochemical function of these complexes is well-documented [49]. Deficiencies in OXPHOS complexes have been related to human disease. Complex I, NADPH dehydrogenase, is encoded by the *Nad* genes. Complex I deficiencies have been associated with LHON, Mitochondrial Encephalomyopathy, lactic acidosis, and stroke-like episodes (MELAS), Leigh syndrome, bipolar disorders, and many other conditions. See Table 1 from [50].

Complex II is composed entirely of nDNA-encoded subunits. Despite being relatively rare, genetic variants to these genes have also been associated with several complex diseases including paraganglioma–pheochromocytomas, a diverse group of renal cell carcinomas, and a specific subtype of gastrointestinal stromal tumors [51,52,53].

Complex III is composed of 13 subunits, one of which is encoded in mtDNA (*MT-CYB*). The principal clinical phenotypes associated with genetic variations in *MT-CYB* are similar to those associated with all other respiratory-chain complexes, but with a particular emphasis on LHON (*G11778A*, *T14484C*, and *G15257A*) [54,55,56,57].

Complex IV, cytochrome c oxidase (*COX*), is encoded by genes *MT-CO1* to *MT-CO3*, and genetic variations in these genes are strongly associated with severe encephalomyopathy and Leigh syndrome (often coupled with cardiopathy, hepatopathy, and nephropathy) [58,59].

Complex V, also known as ATP synthase, is composed of 19 subunits, two of which are encoded in mtDNA (*ATP6* and *ATP8*). Mutations in *MT-ATP6* were the first Complex V genetic mutations to be reported and have since been the most widely documented [60]. Complex V deficiency resulting in pathology is most often associated with m.8993 T > G/C and m.9176 T > G/C mutations (www.mitomap.org, accessed on 1 May 2024). Mutations in *MT-ATP6* have been associated with clinical pictures ranging from isolated ataxia to NARP (Neuropathy, Ataxia, and Retinitis pigmentosa), bilateral striatal necrosis, and Leigh syndrome [61].

#### 2.2.2. Mitochondrial-Derived Peptides

Recent insights into mitochondrial biology have challenged the traditional view of mitochondria as mere powerhouses of the cell, revealing their role in signaling and cellular regulation. Mitochondrial-derived peptides (MDPs), such as humanin, are a class of signaling molecules encoded by short open reading frames (ORFs) within mtDNA [62]. Humanin was first identified in 2001 by screening a cDNA library from neurons of an Alzheimer’s disease patient, marking it as the first known peptide of its kind to be encoded by mtDNA but functionally active outside the mitochondria [63,64,65].

Humanin and other MDPs, including small humanin-like peptides (SHLPs) and Mitochondrial Open reading frame of the Twelve S rRNA type-c (MOTS-c), play important roles in regulating cellular stress responses, apoptosis, and metabolic processes [64,66,67,68]. Humanin interacts with various pro-apoptotic proteins such as BAX and BIM, modulating apoptosis pathways, and has been found in extracellular spaces where it engages with cell-surface receptors such as FPRL-1 and CNTFR/WSX-1/gp130 that are involved in metabolism and inflammation signaling [63,69,70]. 

The discovery of these peptides has expanded the functional repertoire of mtDNA, emphasizing its significance in cellular signaling pathways beyond its traditional role in energy metabolism. The peptides’ locations within the MT-RNR2 and MT-RNR1 genes (encoding the mitochondrial 16S and 12S rRNAs, respectively) suggest a sophisticated level of evolutionary conservation, indicative of their importance in cellular function [62,71,72,73]. Research has shown that variations in these peptides can influence cellular and physiological states, linking mitochondrial genetics directly to disease phenotypes and normal physiological variations [73,74].

As research continues, the role of MDPs in health and disease remains a promising area of study, potentially leading to novel therapeutic targets for diseases characterized by mitochondrial dysfunction and systemic metabolic disturbances. Their inclusion in mitochondrial research provides a broader understanding of the intricate connections between mitochondrial function and overall cellular health [73].

### 2.3. Human mtDNA-Related Diseases

Mitochondrial diseases affect approximately 12.5 out of every 100,000 adults and 4.7 out of every 100,000 children [75]. These conditions demonstrate a bimodal distribution in their onset: an initial peak occurs within the first three years of life, followed by a second, broader peak from late adolescence into the fourth decade, signifying the onset of adult-onset diseases. Notably, symptoms of mitochondrial diseases can manifest even later in life, especially in cases of chronic progressive external ophthalmoplegia (CPEO) [76].

**Figure 2:**
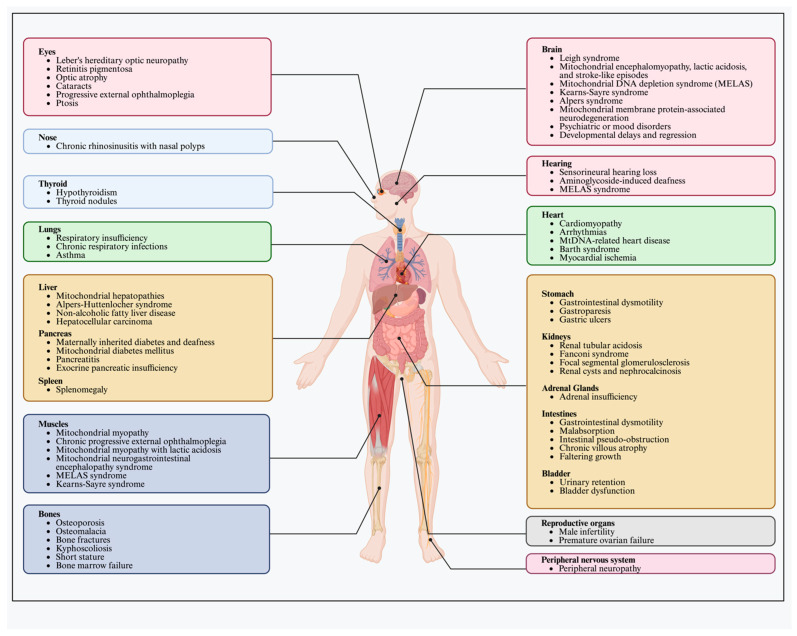
Clinical Manifestations of Mitochondrial Disease. This figure illustrates the organ-specific manifestations of mitochondrial diseases, highlighting affected organs such as the brain, eyes, heart, liver, and musculoskeletal system. Each organ is linked to key mitochondrial diseases that commonly affect it, demonstrating the broad scope of mitochondrial dysfunction.

Diagnosing mitochondrial diseases can present considerable challenges due to their highly variable phenotypes, which can involve virtually any organ system, as depicted in Figure 2. In some instances, the disease may affect a single organ, such as isolated eye involvement in LHON [76]. However, the majority of mitochondrial disease patients exhibit symptoms across multiple systems, underscoring the complex and systemic nature of these disorders (Figure 2).

Diagnosis is further complicated by factors beyond the primary genetic mutations, such as environmental triggers and additional molecular mechanisms that can play critical roles in the disease’s expression and progression. For instance, in LHON, there are significant differences between carriers and affected patients, despite sharing the same mutation.

### 2.4. Specific Particularities of mtDNA in Relation to Human Population History

#### 2.4.1. Uniparental Inheritance

Unlike nDNA, it is widely accepted that mtDNA is inherited maternally, creating clonal or uniparental mitochondrial lineages. This non-Mendelian mode of inheritance has significant implications for our understanding of human population history. During zygote formation, the mtDNA in sperm is believed to undergo degradation, leading to the prevalence of maternal inheritance [77,78,79]. While the precise mechanisms underlying maternal inheritance are not fully understood, hypotheses have been proposed suggesting evolutionary or developmental factors as potential drivers. By exploring the specific particularities of mtDNA inheritance, we can gain valuable insights into the evolutionary dynamics and population history of human populations.

##### Developmental

Due to increased ROS and free radical production as aerobic respiration by-products, sperm mtDNA is highly susceptible to mutations, particularly deletions, which could be harmful to the individual’s development [80,81]. The degradation of sperm mitochondria lowers heteroplasmy levels, thus possibly shielding the offspring from potential pathologies [82]. Therefore, non-transmission of sperm mtDNA to the next generation may pose a developmental advantage. Additionally, this mode of inheritance may have evolved to minimize cytoplasmic mixing, therefore preventing the spread of selfish genomic mutations (i.e., mutations beneficial in terms of mitochondrial replication but deleterious to the host) [83].

##### Evolutionary

According to this hypothesis, there are a few plausible mechanisms behind the lack of paternal mtDNA transmission. Firstly, a simple dilution effect; a single spermatozoon contains 50–75 mitochondria in its midpiece, whereas the mature oocyte is thought to contain between 100,000 and 400,000 [84,85,86]. Secondly, the mitochondrial bottleneck mechanism, whereby mtDNAs replicate from a small number of template mtDNA, magnifies the dilution of minor paternal alleles in early development [87,88]. Although the evolution of such a mechanism may seem counterintuitive, we must consider it in a wider context. Muller’s Ratchet is the process in which there is an irreversible accumulation of deleterious mutations in the genome of an asexual population due to the stochastic occurrence of genetic drift [89,90]. This occurs more in mtDNA than nDNA because of asexual reproduction and because mtDNA is more susceptible to point mutations, indels, and structural changes [91]. The mitochondrial bottleneck mechanism could have therefore perhaps evolved to counteract the effects of Muller’s ratchet.

Furthermore, once sperm enters the oocyte cytoplasm, it is tagged with ubiquitin. This is thought to mark certain sperm components for degradation by the ubiquitin–proteasome system (UPS) [82]. In addition to ubiquitination, the sperm’s mitochondria also undergo autophagy inside the oocyte cytoplasm [92].

##### Paternal Controversy

Despite maternal inheritance being widely accepted as the actual mode of inheritance for mtDNA, there have recently been a few controversial instances where biparental transmission of mtDNA (paternal leakage) was claimed to be observed.

There have been a few reported incidents of paternal transmission. Most prominently, Schwartz et al. reported paternal inheritance in a patient suffering from mitochondrial myopathy [93]. Additionally, Egger and Wilson reported biparental transmission in three families affected with mitochondrial cytopathy [94]. Since the initial suggestion of the possibility of paternal inheritance of mtDNA in humans, no cases were reported despite efforts by several independent groups [95,96,97]. A review by Bandelt et al. established that other instances of biparental inheritance in over 20 publications were likely due to mislabelled samples or contamination [98]. Results such as these have led many in the field to question the validity of these studies and the possibility of paternal inheritance of mtDNA.

More recently, 17 members in three unrelated multigenerational families were found with a high level of mtDNA heteroplasmy [99]. Samples from these individuals were tested by three different laboratories by different technicians using newly obtained blood samples and the results remained consistent. The results in this study were similar to many of the studies discredited by Bandelt et al., posing the question of how many of those studies were dismissed as technical errors and not given the credit they were due.

Conversely, Wei Wei et al. found similar results of perceived paternal leakage; however, upon analysis of the entire nuclear genome sequence, it was established that the leakage was in fact large or unique nuclear mtDNA segments (mega-NUMTs). This study concluded that these rare mega-NUMTs can easily be mistaken for paternal heteroplasmy, thus leading to claims of paternal inheritance of mtDNA [100]. The same results have recently been reproduced by Lutz-Bonengel et al., putting in question the entire paternal inheritance theory [101]. Future studies reporting biparental or paternal mtDNA inheritance should first rule out the possibility of rare mega-NUMTs.

Maternal inheritance is widely accepted as the mode of inheritance for mitochondrial DNA. However, a relatively recent introduction to potential paternal leakage has raised questions [44,45]. We hypothesize that maternal inheritance is indeed the default mode of inheritance and paternal leakage is extremely rare, if at all possible. If biparental transmission should occur, due to the failure of preventative mechanisms, the chances of observing pathology are vastly increased. This raises the question of whether: (i) paternal leakage results in mitochondrial disease, (ii) the detection of paternal leakage may be a product of scrutinizing individuals with known mitochondrial diseases, or (iii) the paternal transmission theory could be the fruit of poorly designed and regulated studies, as well as bias. Any of these hypotheses, if proven, could have implications for association studies of complex traits.

While generally regarded as rare, possibly non-existent, and potentially harmful, paternal leakage of mtDNA might offer certain evolutionary benefits in certain circumstances. Increased genetic diversity, a plausible outcome of paternal mtDNA leakage, could fortify population resilience in the face of environmental changes or adversities, including diseases and climate shifts. Furthermore, if paternal mtDNA harboring benign or advantageous mutations were introduced, this could temper the effects of ‘Muller’s Ratchet’, which is the relentless accrual of deleterious mutations in asexual populations, such as mitochondria [102]. Paternal leakage could introduce less mutated mtDNA into the lineage, potentially tempering this process. Moreover, beneficial mutations, despite their rarity, are a cornerstone of evolution. If a male harbored a favorable mutation in his mtDNA, paternal leakage might serve as a vehicle for this advantage to be passed onto the offspring. While these potential benefits are largely speculative and require rigorous empirical validation, they offer a nuanced perspective on what the role of paternal mtDNA in human health and evolution could be.

The implications of the maternal inheritance of mtDNA and the controversy surrounding the rare reports of paternal leakage extend to genetic studies. Genetic studies are predicated on the assumption of mtDNA’s uniparental inheritance, which allows for certain statistical models that assume all mtDNA variants are inherited from the mother. If paternal leakage occurs, even infrequently, it could introduce confounding variables into these studies, as the mtDNA would not be solely from maternal lineage, leading to potential misinterpretations of mtDNA-associated traits or disease risks.

Furthermore, the occurrence of rare mega-NUMTs could lead to false positives for paternal leakage in genetic studies. Such misidentification could skew the associations between mtDNA variants and certain phenotypes or disease states. If biparental transmission is indeed more prevalent than currently acknowledged, genetic studies may need to adjust their models to account for this additional source of genetic variation. This could have significant implications for the identification of mtDNA haplogroups associated with diseases, potentially altering our understanding of disease mechanisms and inheritance patterns. This further underscores the need for robust genetic validation techniques to discern true mtDNA inheritance patterns, ensuring the accuracy of genetic study interpretations in the complex landscape of human genetics.

#### 2.4.2. Hypermutability

In the absence of recombination (see Section 2.4.6), other evolutionary forces are responsible for mtDNA’s rate of variation. mtDNA is characterized by its hyper-mutability in comparison to nDNA [103,104,105]. In addition to the high presence of elevated ROS generated by the ETC leading to a higher presence of mutations, it has also been proposed that mitochondria could exhibit nucleotide imbalances leading to higher mtDNA mutation rates due to decreased POLG (DNA Polymerase γ) fidelity [106,107]. POLG is a nuclear protein responsible for polymerase activity and recognition and removal of DNA bp mismatches during DNA replication [108]. Further to this, mtDNA is not packaged like nDNA as it lacks protective histones. This has been hypothesized to expose mtDNA to damage that nDNA is spared [109]. However, despite lacking histones, mtDNA is tightly and regularly packed into nucleoids by Mitochondrial Transcription Factor A (*TFAM)*, which may provide at least some protection, although the extent of this protective mechanism remains to be conclusively determined [110].

The hypermutability of mtDNA may have far-reaching implications for genetic analyses. The rapid accumulation of new mutations within a single generation could add an additional layer of complexity to genetic analyses. While rare or de novo mutations are typically excluded from genetic analyses due to their low frequency, in the context of mtDNA, these infrequent mutations can still significantly contribute to disease etiology owing to their rapid accumulation. Moreover, it is our belief that the hypermutability of mtDNA has the potential to confound genetic analyses. In particular, the increased genetic heterogeneity resulting from hypermutability may lead to false associations, challenging the accuracy of genetic study findings, irrespective of which reference sequence is used. Therefore, mtDNA’s hypermutability warrants consideration and tailored analytical approaches in genetic research.

#### 2.4.3. Copy Number and Abundance

In contrast to nDNA, the mitochondrial genome has a varying number of copies within a cell, and this copy number (mtDNA-CN) correlates to its tissue’s bioenergetic needs [111]. It is known that mtDNA-CN varies significantly at different levels including (i) within different mitochondria, (ii) between different cells of the same tissue, (iii) between the same cell types but at different developmental stages, (iv) between different tissues, and (v) between different individuals [112,113]. A mitochondrion contains two to ten copies of mtDNA, and each somatic cell can have up to 1000 mitochondria [2,114,115]. Furthermore, between 1000 and 2000 mitochondria-related genes in nDNA have been identified, which regulate gene expressions and mtDNA copy numbers (mtDNA-CNs) via interactions with mtDNA [116].

Copy number is influenced by genetic factors, with a heritability estimated at 65% [117], indicating the presence of an environmental component too. The relationship of TFAM and mtDNA-CN is an area of active research, and both in vivo and in vitro studies have demonstrated a direct link between TFAM and mtDNA-CN [118,119,120,121]. Animal models have recognized that *TFAM* and other genes play a role in mtDNA-CN levels [113,122]. In fact, animal models have shown that heterozygous ablation of *TFAM* causes a ~ 50% decrease in mtDNA levels [113,118,119], whereas the overexpression of *TFAM* results in a ~ 50% increase in mtDNA copy number [113,119,120,121]. The role of TFAM in mtDNA-CN has recently been underscored in a series of genetic association studies [112,123,124]. However, the intricate control of replication and copy number in mitochondria is a particularity that needs to be considered when designing genetic studies of mtWAS complex traits.

In addition to TFAM, there are other proteins responsible for copy number regulation within the same mechanism, such as TWINKLE and POLG, see Figure 1 from Filograna, Mennuni et al., 2021 [113]. Although our knowledge of mitochondrial genome replication and maintenance has noticeably increased in recent years, the exact functions of some participating elements are unknown. Leading theories on how our cells adjust mtDNA-CN include nucleotide pool availability, ATP requirements, and replication origin regulation [113,125,126,127].

POLG, as previously mentioned, plays a significant role in mitochondrial dynamics. POLG-A (DNA Polymerase γ A), a catalytic subunit of POLG, has been implicated in the regulation of mtDNA-CN. High methylation levels at exon 2 of POLGA were associated with depleted copy number in cells and subsequently associated with differentiation and cancer. POLG-A (DNA Polymerase γ A), a catalytic subunit of POLG, is a key regulator of mtDNA-CN [128].

Copy number has been suggested as a potential biomarker of mitochondrial dysfunction. This is due to its relationship with oxidative stress, energy stores, and mitochondrial membrane potential [129,130]. Additionally, a decrease in mtDNA-CN has been associated with altered cellular morphology, lower respiratory enzyme activity, and reduced essential complex protein expression. Once mtDNA-CN levels are restored, these effects are mitigated [131,132].

Levels of mtDNA-CN can be easily measured from DNA extracted from peripheral blood and other tissues. Peripheral blood extraction remains the most commonly used method due to its accessibility. On account of its low cost and quick results, quantitative PCR (qPCR) has been the most widely used method of measuring mtDNA-CN. However, more recently, studies have shown that whole exome sequencing (WES) and whole genome sequencing (WGS) data are feasible approaches to measuring mtDNA-CN from pre-existing microarrays [123,133,134]. Additionally, seeing as column kit parameters are normally optimized for the isolation of DNA fragments smaller than 50 kB, organic solvent extraction is more accurate than silica-based methods for measuring mtDNA-CN [135]. A potential answer to bias reduction as a result of DNA extraction could be direct measurements as opposed to traditional DNA extraction; for example, measuring mtDNA-CN from direct cell lysis, a process found to be more accurate than both column-based methods and organic solvent extraction [136].

Reflecting on the latest insights from PheWAS and EWAS, the landscape of genetic research is rapidly expanding. A recent PheWAS, analyzing approximately 35,000 samples, illuminates the functional significance of variable number tandem repeats (VNTRs) and multicopy genes. By profiling copy number variations through whole-genome sequencing, the study uncovers 32 traits linked to these structural variations, some of which reside in genomic territories where single nucleotide variant (SNV)-based studies have not identified associations, potentially addressing a portion of the ‘missing heritability’ in genetic research [137]. Another recent PheWAS, leveraging the vast dataset of the UK Biobank, characterizes the phenome-wide effects of CNVs among 472,228 individuals. This study highlights the population-level selection against high-mortality genic loci and pinpoints genic associations with diseases, including syndromic loci at 16p11.2 and 22q11.2, as well as novel variations at 9p23, providing a nuanced understanding of the burden of CNVs across a spectrum of traits [138]. EWAS offer a fresh perspective on mtDNA-CN as a multifaceted biomarker of mitochondrial function. Employing a novel array-based estimation method, ‘AutoMitoC’, across nearly 396,000 UK Biobank participants, a study revealed 71 loci associated with mtDNA-CN and implicates them in diseases such as dementia. This methodological advancement and the ensuing genetic insights underscore the complexity of mtDNA-CN, its regulatory processes, and the causative links to human disease, signaling a step forward in our understanding of mitochondrial genetics and its clinical implications [139].

**Figure 3:**
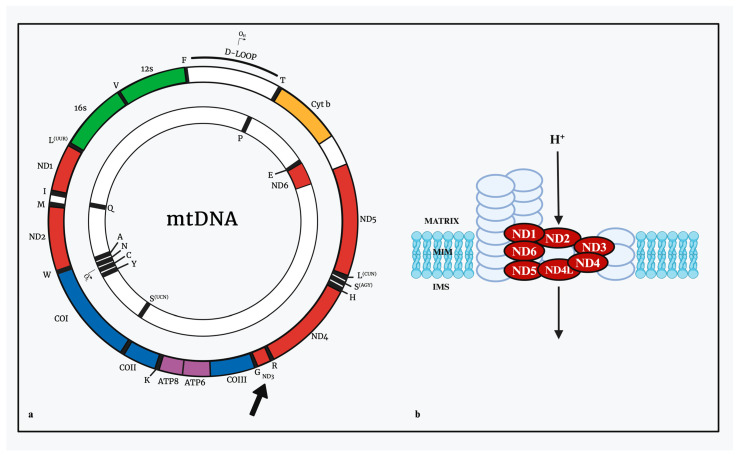
Potential pharmacogenomic effect of variations on mtDNA-CN. This figure illustrates the potential impact of mtDNA-CN variations on pharmacogenomic outcomes. (**a**) Schematic representation of the gene distribution of the seven mitochondrial DNA-encoded subunits of Complex I in the mitochondrial OXPHOS respiratory chain, with a particular emphasis on the ND3 subunit. (**b**) Provides a detailed schematic of Complex I within the mitochondrial OXPHOS respiratory chain. This diagram highlights the ND3 subunit, specifically the amino acid cysteine at position 39 (Cys39), which is the target site for S-nitrosation by the cardioprotective drug mitoSNO. The illustration underscores the potential relationship between mtDNA-CN levels and the efficacy of pharmacological interventions, suggesting that variations in mtDNA-CN might influence the therapeutic effectiveness of drugs that act on mitochondrial components.

In addition to influencing disease susceptibility, insights gained from mtDNA-CN may offer avenues for pharmacological interventions. It is known that certain drugs’ mechanisms of action directly influence mitochondrial complexes. One such example is mitoSNO, a mitochondria-targeted S-nitrosating agent. This drug demonstrates cardioprotective properties in murine models. This agent specifically inhibits complex I through S-nitrosation at Cys39 on the ND3 protein, one of seven mitochondrial DNA-encoded subunits of complex I (Figure 3) [140,141]. Research shows that the efficacies of the drug’s cardioprotective effects were higher in those with increased mtDNA-CN levels [140]. This interaction may suggest a need for further investigation into the potential correlation between mitochondrial abundance in specific tissues and the efficacy of the drug, for if a mechanism is related to the number of complexes in a cell, if mtDNA-CN is higher, then the effect may also be altered. Therefore, future research could investigate strategies that consider mtDNA-CN variations to optimize the therapeutic potential of such interventions.

In genetic analyses, it is critical to consider the wide variation in mtDNA-CN observed among individuals, tissues, and even within cells. This variability has important implications for the interpretation of genetic associations and the identification of causal variants. Copy number alterations in mtDNA have been associated with various diseases and phenotypes, including neurodegenerative disorders and cancer [113]. Accounting for mtDNA-CN variation is important to ensure accurate genetic analyses and to identify potential disease associations. Moreover, environmental factors, such as exposure to toxins, stress, and certain medical conditions, can influence mtDNA-CN by affecting replication and maintenance processes [142]. Understanding the impact of these environmental influences is essential for understanding the relationship between mtDNA-CN and complex traits or diseases in genetic studies. Additionally, the interplay between mtDNA copy number and other mitochondrial parameters, such as heteroplasmy (see Section 2.4.5), is of significance. Changes in mtDNA-CN can impact the relative abundance of mutant and wild-type mtDNA variants, potentially influencing disease manifestation and response to treatments. Therefore, incorporating mtDNA-CN analysis alongside other mtDNA characteristics can provide a more comprehensive understanding of mitochondrial genetics and its implications for complex traits. By considering mtDNA-CN variation, researchers can gain valuable insights into the interplay between mtDNA variation, cellular bioenergetics, and the development of complex traits or diseases.

Mitochondrial abundance refers to the overall quantity or density of mitochondria within a cell. The term is characterized by a degree of ambiguity in the literature, often being used to denote disparate concepts such as the volume or quantity of mitochondria within a cell [143], or even used interchangeably with mtDNA-CN [144]. It is important to acknowledge that, while there is a correlation between these two measures, they are distinct and should be well delineated in scientific discourse. The implications of conflating these terms can be significant, potentially leading to misinterpretations of study results, inaccurate correlations, and compromised comparability between different studies.

#### 2.4.4. Cell-Free Circulating mtDNA (ccf-mtDNA)

In addition to mtDNA-CN, fragments of mtDNA have also been found extracellularly, known as circulating cell-free mtDNA (ccf-mtDNA) [145]. Since its discovery, ccf-mtDNA has been associated with multiple human complex traits [145,146,147,148,149,150,151,152,153]. Our current understanding is that ccf-mtDNA is either released during cell death, as a result of increased metabolic/oxidative stress due to molecular damage, or by active metabolic release from cells [154,155,156]. It can therefore be suggested that ccf-mtDNA may be a biomarker of disease onset and progression. Interestingly, the opposite is seen in Alzheimer’s and Parkinson’s disease (PD); a decreased level of ccf-mtDNA can be observed in cerebrospinal fluid in neurodegenerative disease patients [157]. The role of ccf-mtDNA in neurodegenerative diseases is still somewhat unclear. Further, ccf-mtDNA may hold promise as a biomarker for early stages of neurodegeneration [158].

#### 2.4.5. Heteroplasmy

**Figure 4:**
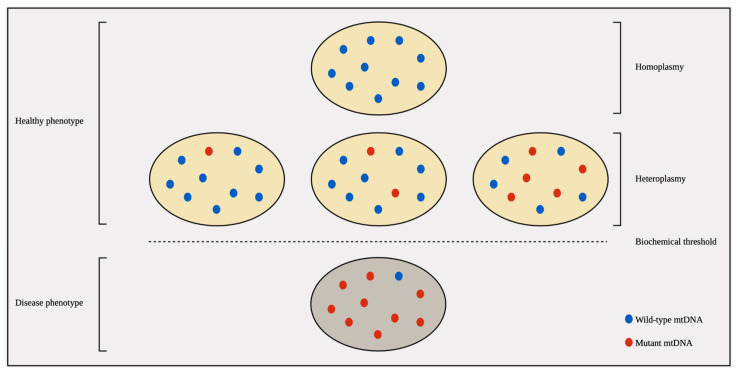
Mitochondrial Heteroplasmy and the Threshold Effect. Cells exhibit tolerance to a high percentage of mutated mtDNA before the manifestation of a disease phenotype. The threshold for deleterious effects is typically above 80%, although it can vary depending on the tissue’s reliance on oxidative metabolism. Adapted from [159].

As previously mentioned, there are multiple copies of mtDNA within each cell. Homoplasmy refers to the state where a mutation in mtDNA is present in all the mtDNA molecules within a cell. When the mutation is only present in a portion of the molecules, this is known as heteroplasmy. The proportion of mutated mtDNA relative to the total determines the heteroplasmy rate. Heteroplasmy levels can vary between cells within the same tissue, different organs in the same individual, and even among individuals within a family. Mutations in mtDNA that occur within approximately three human generations are typically heteroplasmic, resulting in varying proportions of mutated and wild-type mtDNA within a single cell [159]. A further complexity of mtDNA is the threshold effect, wherein a pathogenic mtDNA mutation must reach a critical level within a cell or tissue to manifest deleterious effects on oxidative phosphorylation activity. The threshold is lower in tissues more dependent on oxidative metabolism, further adding to the intricate nature of mtDNA mutations and their functional consequences [160,161]. Cells can tolerate a high percentage of mutated mtDNA before reaching the biochemical threshold that triggers respiratory chain defects, with a typical threshold level exceeding 80% [Figure 4]. This suggests that most mtDNA mutations exhibit haploinsufficiency or a recessive pattern [161]. Further, an individual may exhibit different heteroplasmic levels across different tissues, which is believed to result from random segregation and selection processes [2,34,162,163].

Sequencing techniques that encompass both nuclear and mitochondrial sequences, referred to as nuclear–mitochondrial sequences (NuMTs), can inadvertently complicate the interpretation of heteroplasmy, particularly when employing short-read sequencing methods. This complication arises when sequencing reads from NuMTs are misaligned with the mtDNA reference sequence, leading to an erroneous indication of heteroplasmy. Equally, accurate mtDNA reads may in turn align with NuMT sequences, potentially causing an underestimation of heteroplasmy levels [164,165,166]. To address these challenges, several strategies can be employed. These include the use of long-range PCR for mtDNA amplification, which itself carries a risk of polymerase errors, rigorous quality control to exclude nuclear genome-aligned reads, and a threshold-based approach where heteroplasmies are considered significant only if exceeding a set percentage, commonly more than 1%. However, completely eliminating the impact of NuMTs remains a complex challenge, requiring continued advancements in sequencing and analysis techniques [166,167,168,169].

The presence of heteroplasmy in mtDNA has significant implications for genetic analyses. Careful consideration of heteroplasmy is essential for accurate interpretation and meaningful analysis of genetic data. Firstly, heteroplasmy can affect the identification of causal variants and their functional significance. The varying proportions of mutated and wild-type mtDNA within a cell or tissue can complicate the determination of the specific variant responsible for a phenotype or disease outcome. Accurate attribution of effects to a particular mtDNA variant requires a thorough understanding of the heteroplasmic state and its dynamic nature. Moreover, heteroplasmy can influence disease expression and phenotypic variability. The heteroplasmic rate can impact the severity, age of onset, and progression of mitochondrial disorders. In genetic analyses, accounting for heteroplasmy is crucial for accurately assessing the association between mtDNA variants and complex traits or diseases [159,170]. Failure to consider heteroplasmy can lead to false positive or negative results, misinterpretation of genetic associations, and incomplete understanding of the underlying genetic mechanisms. Furthermore, heteroplasmy exhibits tissue-specific and cell-specific patterns. Different tissues or cells within an individual can harbor varying levels of heteroplasmy, introducing additional complexity to genetic analyses. Tissue-specific heteroplasmy can affect the phenotypic expression of diseases, necessitating the integration of tissue-specific heteroplasmy data in comprehensive analyses [171].

#### 2.4.6. Haplogroups

Mitochondrial haplogroups, representing collections of similar haplotypes, are defined by distinct combinations of SNPs in mtDNA differing from reference sequences, each tracing back to a shared ancestor [172]. The formation of these haplogroups results from the sequential accumulation of mutations along maternal lineages, a process analogous to the paternal lineage accumulation in Y-chromosome lineages [11]. This contrasts with nuclear DNA mutations, which are inherited from both parents. The current, most comprehensive phylogenetic tree for human mitochondrial genomes encompasses 6401 unique haplogroups [173,174]. The mutations that have occurred throughout human history have led to the subdivision of mtDNA into discrete haplogroups, often with distinct regional affiliations. For instance, 90% of the European population is encompassed within five major mitochondrial haplogroups [175,176].

The D-loop, as the hypervariable region within the mitochondrial genome, plays a critical role in haplogroup derivation. This classification of mtDNA haplogroups can be achieved through the use of algorithms used in several tools including Haplogrep3 [174], MitoMaster, [177] MitoTool [178], Phylotree 17 [179], and HaploFind [180]. These tools base the inference of haplogroups on their choice of phylogenetic tree. These tools have undergone significant improvements over the years to overcome common pitfalls associated with haplogroup classification. Some of these pitfalls include heterogeneous input data sourcing (e.g., genotyping arrays, WGS samples, Sanger-sequenced data, long-read data, etc.) requiring systematic quality control of the input data, critical to maintaining data integrity (See (Schönherr, Weissensteiner et al., 2023) and references therein) [174]. Further, phylogenetic trees have been updated over the years to improve haplogroup classification [179].

Haplogroup distribution across the global population varies geographically. This has been previously attributed to genetic drift, specifically the colonizing of new geographic regions by only a few immigrants that contributed to a limited number of mtDNAs [181]. The evolution and migration of the ancestral clades, originating in Africa, have been well documented [182,183]. From Africa, haplogroups diverged into all continents, giving rise to the haplogroup variation we now observe in global populations [184]. More recently, alternative hypotheses are also being suggested to potentially play a role, including natural selection due to climate differences. It has been suggested that functional mtDNA variants may have conferred selective advantages by altering the allocation of calories between ATP production for work or for heat production, thereby facilitating adaptation to extreme cold. Mitochondrial variants can impact mitochondrial coupling efficiency. Consequently, natural selection would favor the enrichment of regionally appropriate mitochondrial coupling efficiency, leading to the emergence of distinct mtDNA haplogroups that align with specific geographical regions [181,185,186,187,188].

The phylogenetic clustering of mtDNA haplogroups has been reported to be specific to populations in Africa [189,190], Asia [191,192,193,194], Europe/Eurasia [195,196], and Native America [197]. Particularly, there are nine major haplogroups among Europeans (H, U, J, T, K, W, I, V, and X) [196], while there are four major haplogroups in African populations; L0, L1, L2, and L3 [198]. For continental-level studies, one should consider the primary haplogroups associated with each continent’s native populations. Although there are limited contradictions in the literature regarding the haplogroups associated with each continent’s native population, this must be considered when designing population studies as it may disrupt interpretation.

The initial evidence suggesting a link between mtDNA haplogroups and disease predisposition came from the observation that European haplogroup J increases the penetrance of milder mutations in Leber hereditary optic neuropathy (LHON) [199,200,201]. Since then, there have been several studies associating different haplogroups with different diseases [184,188,202,203,204].

Haplogroups represent genetic subgroups that can exhibit different frequencies of disease-related genetic variants and different genetic backgrounds. Lack of replication studies for previously positive association studies may result in false associations. An indication of this would be that the initial association is not replicated for the same haplogroup in a second sample. It is possible that the reason for the first association could be that there was an overrepresentation of a causal variant in the first sample, leading to the erroneous association made between the disease and the haplogroup. By including haplogroup information as a covariate, and therefore obtaining a random sample, researchers can minimize confounding effects and improve the accuracy and robustness of their findings.

In the context of genetic analyses, incorporating haplogroups can provide additional insights into the genetic architecture of complex traits and diseases. Haplogroups can interact with nuclear genetic factors and influence the phenotypic expression of diseases [205]. By examining haplogroup-specific effects and gene–gene interactions, researchers can explore the complex interplay between mitochondrial and nuclear genomes, expanding our understanding of disease mechanisms. Moreover, haplogroups can be used in fine-mapping studies to identify regions of interest and potential causal variants. By leveraging the phylogenetic structure of haplogroups, researchers can prioritize specific genetic regions for further investigation, enabling more targeted and efficient genetic analyses [205].

With the prevailing maternal inheritance theory, the lack of recombination in mtDNA further shapes its genetic landscape and warrants careful consideration in genetic analyses. Unlike nDNA, mtDNA’s maternal inheritance means it undergoes negligible intermolecular recombination at the population level. As a result, mtDNA lineages are largely considered to be clonal [206]. This may have important implications for population stratification and fine-mapping of causal variants.

Linkage disequilibrium (LD) refers to the non-random association of alleles at different loci in a given population. While previous studies have reported on the differences in LD patterns between mtDNA and nDNA [182], there remains a lack of comprehensive evaluation of mtDNA LD, particularly using WGS data. This gap is particularly notable in populations outside of European descent [182].

#### 2.4.7. Lack of Introns

The absence of introns in mtDNA is a unique characteristic that warrants careful consideration in genetic analyses. Unlike nDNA, which contains non-coding intronic regions involved in gene regulation and alternative splicing, mtDNA lacks introns, resulting in a simplified genetic structure [207,208].

As discussed in Section 2.1.1, other than the NCR, the entire mitochondrial genome consists of coding DNA. This means that most of its sequence is used to produce proteins, unlike nDNA, which contains a large proportion of non-coding DNA, including introns. Therefore, mtDNA mutations are more likely to have functional consequences, potentially affecting mitochondrial function [209].

Although this review primarily focuses on the implications of mtDNA in genetic studies, it is important to acknowledge that the unique structure of mtDNA, notably its intron-less nature, makes it a valuable asset in various other genetic research fields. The distinct features of mtDNA render it particularly suitable for a range of studies, including forensic analysis, phylogenetic investigations, personal identification, population genetics, functional genomics, mitochondrial proteomics, and pharmacogenomic studies, as well as evolutionary biology and ecology. For instance, the design of functional genomic studies can be influenced by mtDNA’s intron-less nature requiring alternative primer design strategies and a concentrated focus on specific coding regions. Conversely in nDNA, the presence of introns enables the design of primers targeting specific exon–intron boundaries, facilitating the analysis of specific gene regions.

The implications of this lack of introns in mtDNA are multifaceted and impact the interpretation and analysis of genetic data. Firstly, the absence of introns simplifies gene expression regulation within the mitochondria. Transcription factors and epigenetic modifications assume critical roles in governing gene expression in mtDNA [110]. Consequently, understanding these alternative regulatory mechanisms becomes important when investigating associations between mtDNA variants and complex traits or diseases.

#### 2.4.8. Sensitivity of mtDNA to Environmental Factors

Mitochondrial DNA further differs from nDNA through its sensitivity to environmental factors. The proximity of mtDNA to the ETC exposes it directly to reactive oxygen species (ROS), leading to increased susceptibility to oxidative damage [210]. In contrast to nDNA, which benefits from extensive protective and repair mechanisms, mtDNA lacks comparable safeguarding histone proteins and has limited DNA repair capabilities. This inherent vulnerability of mtDNA to environmental stressors, including oxidative stress, chemical toxins, and ionizing radiation, has significant implications for understanding mitochondrial pathologies and the broader impact of environmental factors on cellular function and genomic stability [109].

Environmental pollutants can significantly impact human health, and their effects on mitochondrial function have gained attention in the field of toxicology. MtDNA is directly exposed to environmental stressors, including oxidative stress, toxins, and nutritional factors, which can induce mitochondrial dysfunction and lead to the generation of deleterious mutations or alterations in mtDNA-CN [211]. Mitochondria, as central hubs of mechanistic endpoints, are increasingly recognized as key organelles targeted by environmental contaminants [212]. Contaminants such as polycyclic aromatic hydrocarbons, pesticides, and persistent organic pollutants have been associated with mitochondrial dysfunction, altered mitochondrial dynamics, and disruption of metabolic pathways [213].

Mitochondrial dysfunction induced by environmental pollutants can have significant implications for disease susceptibility and development. The alteration of mitochondrial respiratory chain complexes, ATP depletion, and increased oxidative stress are among the mechanisms involved [214]. Numerous studies have linked environmental contaminants to metabolic diseases, including obesity and type 2 diabetes [215,216,217]. For instance, the herbicide atrazine has been associated with increased obesity risk, while benzo(a)pyrene and pyrethroids have been linked to metabolic reprogramming and neurodegeneration, respectively.

Furthermore, environmental pollutants can disturb the crosstalk between mitochondria and epigenetic marks. Mitochondrial metabolism plays a crucial role in providing metabolites involved in epigenetic patterning, such as acetyl-coA, NAD+, NADH, and S-adenosylmethionine. Changes in the availability of these metabolites can affect histone acetylation, DNA methylation, and the activity of epigenetic-modifying enzymes [218]. This disruption of epigenetic patterns can have consequences on gene expression and cellular function, contributing to the development of various diseases, including cancer [219].

Considering the impact of environmental pollutants on mitochondrial function and their interaction with the epigenome is critical in genetic analyses. Integrating mitochondrial data, epigenetic information, and environmental exposures in these analyses allows for a more comprehensive understanding of disease susceptibility and etiology. By incorporating these factors, researchers can gain insights into the complex interplay between genetics, environmental exposures, and mitochondrial responses, leading to a better understanding of disease mechanisms and the development of personalized medicine approaches.

#### 2.4.9. Epigenetics

Mitochondrial DNA is susceptible to epigenetic changes through various mechanisms [220]. One such mechanism is DNA methylation, the addition of a methyl group to cytosine, which is an epigenetic mechanism that has been widely studied [220,221,222,223]. While methylation does occur in mtDNA, it occurs at significantly lower rates than in nDNA, ranging from one-fourth methylated to one-fourteenth methylated compared to nDNA [221,224]. Other epigenetic mechanisms include non-coding RNA. These epigenetic mechanisms can impact mtDNA directly through mtDNA genes, or through nuclear genes involved in mitochondrial processes [220].

The communication between nuclear and mitochondrial DNA is bidirectional. This is critical to maintaining cellular homeostasis and ensuring functionality. This interaction between the nucleus and mitochondria occurs at several different stages, including protein–protein interactions in OXPHOS, nuclear factor–mtDNA recognition site interactions in replication and transcription, and protein–RNA interactions [225]. The interplay between nDNA and mtDNA epigenetic modifications can have significant implications for gene regulation and the manifestation of complex traits and diseases [109]. Epigenetic modifications in mtDNA can potentially affect nuclear gene expression through retrograde signaling pathways and influence cellular metabolism, energy production, and oxidative stress responses [226].

Considering the epigenetic crosstalk between mtDNA and nDNA is critical in genetic analyses, offering insights into how epigenetic modifications in mtDNA modulate nuclear gene expression and contribute to disease susceptibility. This understanding aids in elucidating underlying molecular mechanisms and pathways involved in complex traits. Moreover, the integration of mtDNA epigenetic information can refine the interpretation of genetic study results. By incorporating epigenetic profiles into the analysis, researchers can identify potential regulatory regions, non-coding RNAs, or other functional elements within mtDNA that contribute to disease phenotypes. This knowledge can lead to the discovery of novel disease-associated variants and enhance the accuracy of genetic risk prediction models. Furthermore, understanding the epigenetic crosstalk between mtDNA and nDNA creates opportunities for personalized medicine approaches. By considering individual-specific epigenetic profiles, it becomes possible to tailor interventions, therapies, and preventive strategies to account for the unique mitochondrial and nuclear genomic interactions and their impact on disease susceptibility and treatment response.

Epigenome-wide association studies (EWAS) explore associations between epigenetic markers and phenotypic traits, advancing therapeutic and diagnostic approaches. EWAS integration with genetic studies provides comprehensive insights into genetic and epigenetic components, improving understanding of disease etiology and pathogenesis s [227,228].

## 3. Conclusions

Mitochondria are complex organelles. There are significant particularities that set mtDNA apart from the nuclear genome, warranting consideration when designing and carrying out genetic analyses. Since the initial identification of mitochondria and its genome, substantial progress has been made in understanding their structure and function. However, this understanding is challenged by the holistic nature of mitochondrial operations: disruptions in one aspect of mtDNA, including mutations, haplogroups, mtDNA-CN variations, or ccf-mtDNA, invariably affect other aspects. This interconnectivity requires an integrated approach to mitochondrial research, as focusing on isolated elements can lead to a fragmented understanding. The future of mitochondrial research interpretation might be greatly advanced by the development and application of artificial intelligence (AI) and novel analytical algorithms to overcome the limitations highlighted in this article. These technologies offer the potential to synthesize and contextualize disparate mitochondrial data effectively, enabling a more comprehensive correlation of findings across various aspects of mitochondrial mechanics. Embracing these integrated approaches is essential for advancing our understanding of mitochondrial biology in its entirety.

## Figures and Tables

**Table 1:** Distribution of Genes Encoded on the Heavy and Light Strands of Mitochondrial DNA.

GENES ENCODING	HEAVY STRAND	LIGHT STRAND
**rRNA**	12s (*MT-RNR1*)	
	16s (*MT-RNR2*)	
**tRNA**	*MT-TR*	*MT-TA*
	*MT-TD*	*MT-TC*
	*MT-TG*	*MT-TE*
	*MT-TH*	*MT-TN*
	*MT-TI*	*MT-TP*
	*MT-TLI* (*CUN*)	*MT-TQ*
	*MT-TL2* (*UUR*)	*MT-TS1* (*UCN*)
	*MT-TK*	*MT-TY*
	*MT-TM*	
	*MT-TF*	
	*MT-TS2* (*AGY*)	
	*MT-TT*	
	*MT-TW*	
	*MT-TV*	
**POLYPEPTIDES**	**COMPLEX I**	**COMPLEX I**
	*MT-ND1*	*MT-ND6*
	*MT-ND2*	
	*MT-ND3*	
	*MT-ND4*	
	*MT-ND4L*	
	*MT-ND5*	
	**COMPLEX III**	
	*MT-CYB*	
	**COMPLEX IV**	
	*MT-CO1*	
	*MT-CO2*	
	*MT-CO3*	
	**COMPLEX V**	
	*MT-ATP6*	
	*MT-ATP8*	
**TOTAL GENES**	**28**	**9**

## Data Availability

No new data were created or analysed in this study. Data sharing is not applicable to this article.
